# The temporal changes in GPT-4 performance on UKMLA practice questions: educational and clinical implications

**DOI:** 10.3389/fmed.2026.1888765

**Published:** 2026-07-21

**Authors:** Ravanth Baskaran, Sai Sirikonda, Aditya Singh, Sripradha Srinivasan, Becky Leveridge, Octavi Casals-Farre, Harmeena Kaur, Susruta Manivannan, Kabilan Elangovan, Chrystie Wan Ning Quek, Kanae Fukutsu, Judith Cave, Bhaskar Kumar Somani, Daniel Shu Wei Ting, Athanasios Hassoulas

**Affiliations:** 1 University Hospital Southampton NHS Trust, Southampton, United Kingdom; 2 Centre for Medical Education (C4ME), School of Medicine, Cardiff University, Cardiff, United Kingdom; 3 University Hospitals of North Midlands NHS Trust, Stoke-on-Trent, United Kingdom; 4 Division of Gastrointestinal and Hepatobiliary Surgery, Imperial College Healthcare NHS Trust, London, United Kingdom; 5 Hawke’s Bay Hospital, Hastings, New Zealand; 6 Department of General Medicine, Doncaster Royal Infirmary, Doncaster, United Kingdom; 7 Singapore Eye Research Institute, Singapore National Eye Centre, Singapore, Singapore; 8 Artificial Intelligence Office, Singapore Health Services, Singapore, Singapore; 9 Department of Ophthalmology, Faculty of Medicine and Graduate School of Medicine, Hokkaido University, Sapporo, Japan

**Keywords:** artificial intelligence, GPT-4, machine learning, medical education, UKMLA

## Abstract

**Background:**

Generative Artificial Intelligence (GAI) models, such as GPT-4, have been extensively studied for their integration into medical practice and education. GPT-4 has demonstrated excellent performance on medical licensing examinations, including the United Kingdom Medical Licensing Assessment (UKMLA). However, the field lacks longitudinal data on whether such performance is stable or varies over time. Theoretically, iterative improvements updated by the vendor should enhance performance, but empirical evidence of such longitudinal trends remains limited. Given that GPT-4 undergoes periodic vendor-side updates, we aimed to analyse the categorical, time-spaced performance of GPT-4 on the UKMLA to characterise how its performance changes over time in a medical context.

**Methods:**

Two publicly available UKMLA papers were fed into GPT-4 at two different time points, June 2023 and December 2024. 191 questions were provided with and without multiple-choice options to assess GPT-4’s clinical competence. McNemar’s test was performed to evaluate changes in GPT-4’s performance over time, comparing domain-specific questions.

**Results:**

GPT-4’s accuracy improved noticeably between the two rounds (MCQ: 88.0 to 93.7%, *p* = 0.027; non-MCQ: 68.1 to 81.7%, *p* = 1.00). Single-step accuracy rose from 73.1 to 82.3%, and multi-step from 57.4 to 80.3% without MCQ. GPT-4 showed improved accuracy from Round 1 to Round 2 for both single-step and multi-step questions, with MCQ-prompted responses consistently outperforming non-MCQ responses (up to 95.1% accuracy for multi-step MCQ questions in Round 2). GPT-4’s performance improved across all question categories from round one to round two, most notably in management questions without MCQ options (+23.30%), though these differences were not statistically significant.

**Discussion and conclusion:**

GPT-4’s performance on the UKMLA improved significantly over 18 months, suggesting that iterative vendor-side model updates enhance clinical reasoning capabilities. These findings indicate that GPT-4 may serve as a supplementary educational tool for medical students and clinicians; however, the underlying drivers of performance changes remain opaque, and such tools should be deployed with structured oversight to prevent overreliance.

## Introduction

The rapid advancement of technology has resulted in what Alan Turing described as thinking machines, Generative Artificial Intelligence (GAI) (1). These GAI utilise neural transformer models that can process vast amounts of data, enabling the creation of complex natural language processing and coherent, constructive output (2, 3). OpenAI has not publicly disclosed the parameter count or training data volume for GPT-4; however, we know GPT-3 was trained on 175 billion parameters using approximately 570 gigabytes of publicly available online information. The scale of GPT-4’s architecture likely substantially exceeds that of GPT-3, based on observed performance capabilities and architectural innovations, though exact specifications remain proprietary (4–8).

GPT-4’s ability to sift through large volumes of backend vector datasets and provide coherent, logical, and structured responses sparks considerable excitement about AI’s role in clinical practice and medical education (9, 10). The model could be used as a virtual teaching assistant, providing instant, 24/7 feedback and reducing educators’ time and resource demands (11, 12). It could also be utilised to personalise curriculum plans tailored to an individual’s learning needs, ensuring that information is communicated and understood effectively by the end user (13, 14). In a clinical setting, GPT-4 could be used as a decision-aiding tool for clinicians, streamlining pathways and providing evidence-based suggestions (15, 16). Early studies analysing the performance of GAI in answering questions about chronic conditions, gastro-oesophageal reflux, and colorectal cancer demonstrate that GAI can also serve as a patient information source, reducing clinicians’ burden and providing an interactive resource for patients (17, 18).

However, the field lacks longitudinal data on whether such performance is stable or varies over time. Furthermore, many studies have highlighted variability in performance and reliability, including the potential to produce incorrect information or inaccurate explanations of medical concepts, a phenomenon known as hallucinations (19, 20). Despite this, further studies have shown that improvements in newer GAI models have enhanced their clinical capabilities (21, 22).

If GPT-4 is to be trusted as an information source by students, clinicians, and patients, it is vital that its performance be regularly evaluated to enable ongoing improvement and to identify changes over time. Medical licensing exams provide a broad, standardised benchmark for comparing GAI performance, and many studies have assessed GAI performance on these tests (23). Studies by Gilson et al. (24) and Kung et al. (12) demonstrated that GPT-4 exhibited knowledge and skills that surpassed those of the average learner on the United States Medical Licensing Examination (USMLE), with high levels of concordance and insight into its abilities and explanations. Similarly, GPT-4 has exceeded the minimum competency threshold, as demonstrated by the German medical board (25). Furthermore, studies by Casals-Farre et al. (26) and Lai et al. (27) revealed that GPT-4 performs exceptionally well on the United Kingdom Medical Licensing Assessment (UKMLA), passing both exams with high scores.

Theoretically, with the exponential increase in the amount of data on the internet feeding into LLMs’ datasets, natural language processing models such as GPT-4 should be able to adapt and enhance their knowledge (28–30). In this study, we evaluated GPT-4’s temporal performance on the UKMLA through a categorical analysis of questions and their curated answers to assess improvements in the model over an 18-month period.

## Methodology

### Question source

Sample questions were obtained from the Medical School Council (MSC) website (31). The website was first accessed on June 10th, 2023, and then again on December 16th, 2024. The first iteration (2023) of mock questions available on MSC’s website was used throughout this study.

Twenty-four areas of clinical practice are tested across two papers in the UKMLA Applied Knowledge Test (AKT) (28). Each paper consists of 100 questions, including nine image-based questions in the 2023 iteration. Each question includes a question stem and five multiple-choice options. In our study, 191 questions were included because GPT-4 did not process images when its performance was first assessed in 2023. To standardise our data, we excluded these image-based questions from both analyses.

### Model and testing environment

Questions were administered via the consumer ChatGPT web interface^1^ using a ChatGPT Plus subscription, with the model selector set to “GPT-4”. As the consumer interface does not expose sampling parameters, temperature, top-p and maximum token settings were left at their platform defaults and could not be modified. No system prompt or custom instructions were configured. The memory feature was disabled, and no plugins, tools, file uploads, or advanced data analysis were used; web browsing was enabled. Each question was entered into a new, fresh chat session with no prior conversation history, and the chat was closed before the next item to prevent context carryover. Each question was submitted once (a single attempt) (Table 1).

**Table 1:** Model configuration and testing environment parameters.

Parameter	Round 1
Testing dates	Round 1: 10–15 June 2023; Round 2: 16–19 December 2024
Exact model	GPT-4 (both rounds)
Access route	Consumer ChatGPT web interface (chat.openai.com)
Model selector setting	GPT-4
Web browsing	On
Plugins/tools	None
File uploads/Advanced data analysis	Not used
Memory feature	Disabled
System prompt/custom instructions	None (left blank)
Temperature	Platform default; not user-modifiable
Top-p/maximum tokens	Platform default; not user-modifiable
Prompt wording – non-MCQ	Question stem only (clinical scenario + leading questions)
Prompt wording – MCQ	Question stem + all five answer options
Attempts per questions	1
Conversation reset	New, fresh chat per input; chat closed before next input

### Large language model (LLM) inputs

Each paper was evaluated separately. The question stem, including the scenario and leading question, was first input into GPT-4 without answer options, and this response was then recorded under the “non-MCQ” section. The entire question, along with five MCQ prompts, was subsequently entered into a separate, fresh chat, and the response was recorded under the “MCQ prompt” section. The identical GPT-4 configuration described above was used on both occasions, enabling standardisation. The model was not primed with any preliminary information on either occasion to recreate the typical day-to-day use of a medical student/clinician.

### Data collection

The data were collected between 10/06/2023 and 15/06/2023 (Round 1) and again between 16/12/2024 and 19/12/2024 (Round 2) using the same methods at both time points, enabling longitudinal comparison across an approximate 18-month interval.

The accuracy of answers provided by the GPT-4 was coded as per the following categories:

1. Correct: the answer provided is entirely correct and correlates with the marking scheme.
2. Incorrect: the answer provided is entirely inaccurate and does not adhere to the marking scheme.
3. Indeterminate: the answer provided is neither entirely accurate nor entirely inaccurate.

One author, a medical doctor (SS), independently determined the answer category, and any queries were assessed separately by a senior author. These were determined at the beginning of the study, during the first iteration of data collection, using the same categories used subsequently.

#### Non-MCQ condition

The non-MCQ condition presented the question stem alone, without answer options. This format represents a free-response approximation of the UKMLA task, which is designed as a single-best-answer MCQ. Free-text responses were evaluated against the official mark scheme using the three-category rubric above. A response was classified as correct if it identified the correct answer, whether stated alone or embedded within additional reasoning or detail. A response was classified as indeterminate if it demonstrated partial understanding or provided a clinically reasonable answer that did not identify the correct answer. All other responses were classified as incorrect. Supplementary Table 1 provides examples of our scoring rubric.

### Question categorisation

Questions were initially categorised by complexity based on the number of logical steps needed to solve them. A question was classified as either single-step or multistep. A question was classified as either a single-step question (requiring one logical deduction) or a multistep question (requiring two or more stages of analysis). For example, a multistep question might require diagnosing a condition based on the clinical presentation and then selecting a second-line treatment due to contraindications to the first medication. One author (SS), a medical doctor, independently determined the complexity category in a blinded manner. Any queries were resolved by the senior author. These were determined at the beginning of the study, during the first iteration of data collection, using the same categories used subsequently.

GPT-4’s domain-specific strengths were then analysed by assessing its performance in different competencies. We categorised questions into five broad competencies as follows:

1. Diagnosis: identifying the underlying cause of clinical presentation.
2. Investigation: choosing the most appropriate test from bedside lab tests to imaging within the context of the clinical vignette.
3. Core medical knowledge: applying essential principles of anatomy, physiology, and biochemistry.
4. Management: choosing the most suitable treatment plan within the context of the clinical vignette.
5. Pharmacology/Prescribing: understanding drug actions, adverse reactions, contraindications, and therapeutic prescribing.

Each question was independently categorised into one of the competency domains by a medical doctor (SS). Queries were resolved by a senior author. Because some questions are frequently mapped to multiple competency domains, we applied a hierarchical rule: the competency domain deemed most relevant and critical to selecting the correct answer. This approach ensured mutually exclusive categorisation, enabling unambiguous domain-specific analysis. Categories were determined at the beginning of the study, during the first iteration of data collection, and were used subsequently.

Overall question accuracy was observed both with and without MCQ prompts. These were then analysed in terms of question complexity and assessed competency. All results were analysed using IBM SPSS version 29, with descriptive statistics and McNemar’s test.

## Results

Our primary hypothesis predicted improved overall accuracy in GPT-4’s performance when attempting the UKMLA mock during the second round of data collection.

### Overall performance

During the initial data collection phase (June 2023), GPT-4 achieved a total score of 88.0% (168/191) across both papers one and two with MCQ options. This score dropped to 68.1% (130/191) when MCQ options were not provided. In the second phase (December 2024), GPT-4’s overall performance improved to 93.7% (179/191) for MCQs and 81.7% (156/191) for the non-MCQ set. A McNemar test comparing the correlation between “non-MCQ” and MCQ performances across both time points. For crosstabulation during the McNemar test, all indeterminate responses were counted as incorrect. Hence, the two categories used were “correct” and “incorrect”. Comparing “non-MCQ” responses across both time points, the test showed a *p-*value of 1.00, indicating statistical non-significance. Comparing overall performance in the “MCQ” sections between the first and second phases showed a statistically significant difference (*p* = 0.027).

No indeterminate questions were answered on either occasion when MCQ prompts were provided. The level of indeterminacy decreased between the two data stages when no MCQs were provided, with 19 indeterminate answers in the first stage and 12 in the latter.

### Step analysis

**Figure 1:**
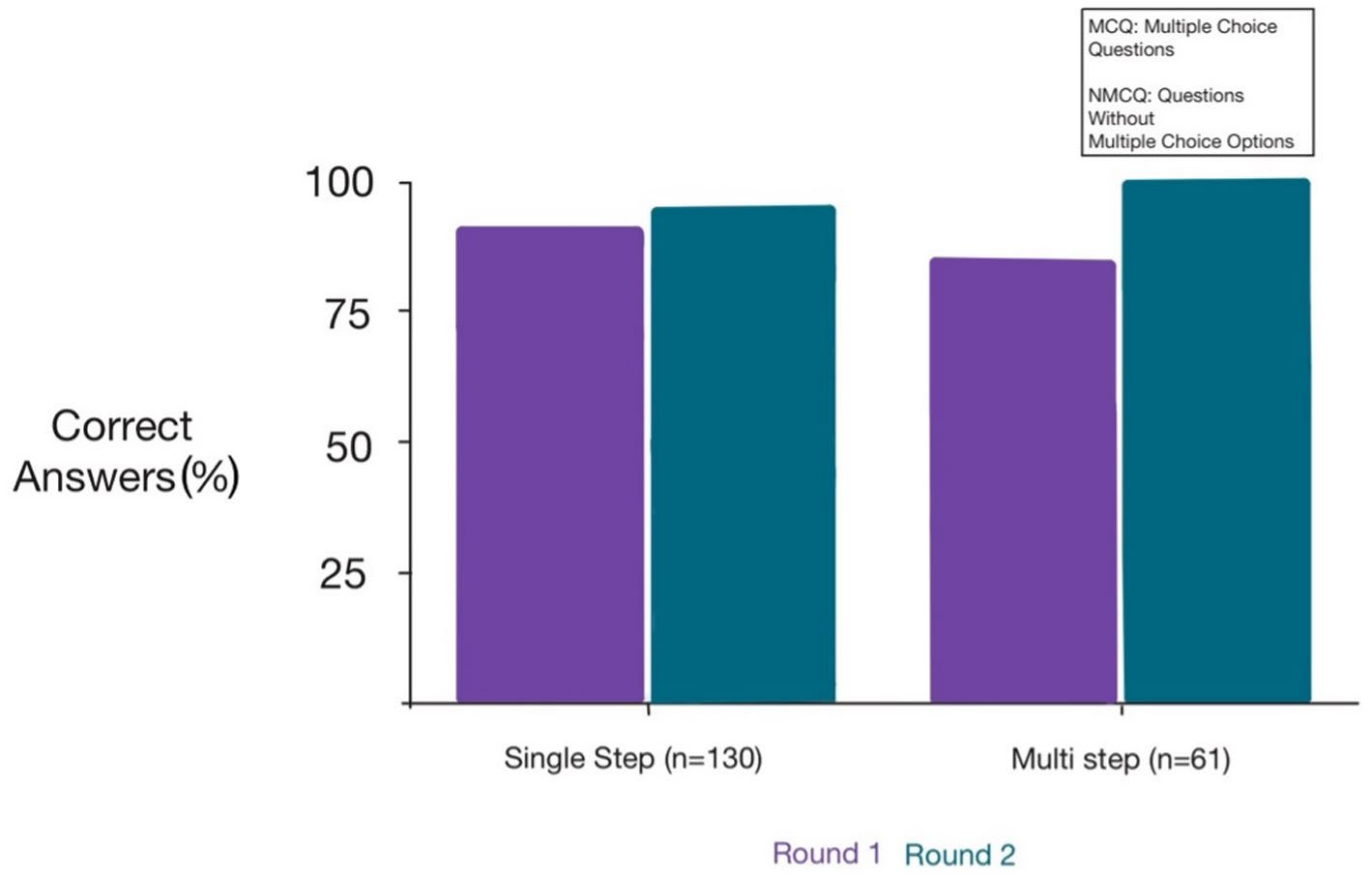
GPT-4’s performance in single-step and multistep questions with the MCQ options provided.

**Figure 2:**
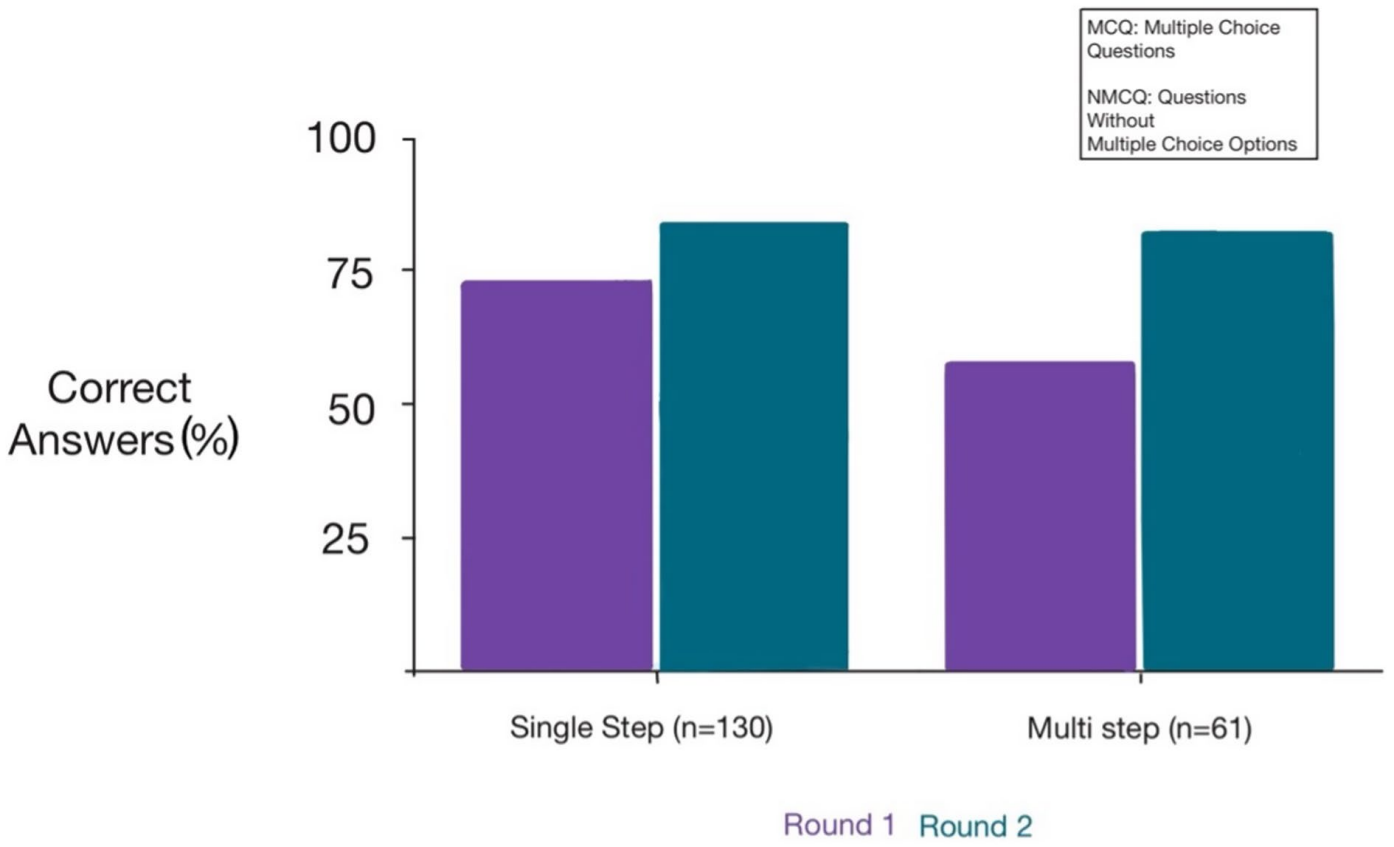
GPT-4’s performance in single-step and multistep questions without the MCQ options provided.

We calculated a total of 130 single-step and 61 multi-step questions across both papers. During the first round, GPT-4 answered 95/130 (73.1%) single-step and 35/61 (57.4%) multi-step questions without MCQ options. This improved to 117/130 (90.0%) and 51/61 (83.6%) correct single- and multi-step questions with MCQ prompts, respectively. During the second round, GPT-4 answered 107/130 (82.3%) single-step and 49/61 (80.3%) multi-step questions without MCQs; and 121/130 (93.1%) single-step and 58/61 (95.1%) multi-step questions with MCQs (Figures 1, 2).

### Competence analysis

There were 57 diagnoses, 27 investigations, 32 core medical knowledge, 43 management, and 32 pharmacology questions.

Management questions showed the greatest differences between the two rounds, without the MCQ option. In diagnosis questions, when provided with MCQ prompts, GPT-4 performed 5.30% better and 7.02% better without MCQ options in the second round. The most notable improvement was seen in “management” questions without MCQ, where GPT-4’s performance increased by 23.30% in round two. The least improvement occurred in “management” and “core medical knowledge” questions with MCQ options, with only a 2.30 and 3.10% improvement, respectively.

GPT-4 scored 100% in round two of “investigation” questions when MCQs were provided. The lowest score was in round one “management” questions, where no MCQ prompts were given, with GPT-4 scoring 51.20%.

**Figure 3:**
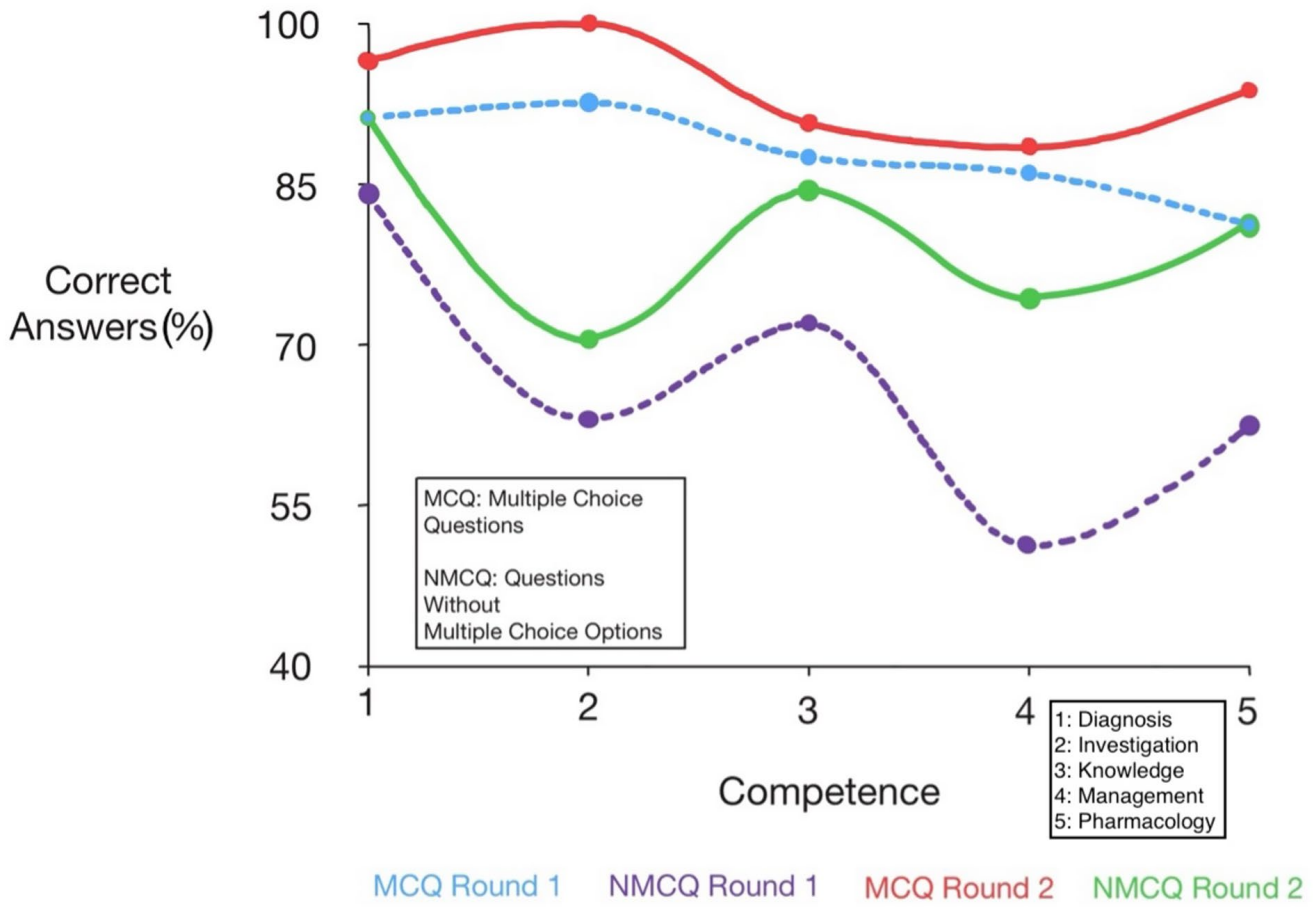
GPT-4’s performance across all five competencies in rounds one and two with and without MCQ options.

Figure 3 illustrates the competence-specific performance trends across both prompting rounds. The “MCQ Round 2” line shows the highest accuracy across all competencies, ranging from 88 to 100%. This is followed by “MCQ Round 1” (92–92%), “Non-MCQ Round 2” (74–90%) and finally “Non-MCQ Round 1” (50–83%). The most pronounced gain in accuracy is seen in “management” questions under non-MCQ conditions, where a marked trough from round 1 (51.2%) shifts upwards to 74.5% in round 2, aligning with the reported 23.3% improvement. GPT-4 shows high accuracy in diagnosis and pharmacology in round 1 and continues to increase in round 2. Core medical knowledge and management questions show the smallest gains in MCQ-related performance between the two rounds. Overall, the curves illustrate the largest improvements in free-response answers (non-MCQ) between the two rounds.

Statistically significant results based on McNemar testing are highlighted in Table 2, with *N* = 191 and α = 0.05 for all values (Tables 2, 3).

**Table 2:** The overall performance of GPT-4 through both rounds of data collection.

	ROUND 1		ROUND 2	
	Without-MCQ options	With MCQ options	Without-MCQ options	With MCQ options
Correct	130	168	156	179
Incorrect	42	23	23	12
Indeterminate	19	0	12	0

**Table 3:** GPT-4’s performance on each competency in each round, along with the overall change between the two rounds, as assessed using the McNemar test.

	With MCQ				Without MCQ			
	Round 1(R_1_)	Round 2 (R_2_)	△_1_ (R_2_-R_1_)	*p-*value	Round 1(R_3_)	Round 2 (R_4_)	△_2_ (R_4_-R_3_)	*p-*value
Diagnosis (*n* = 57)	91.20%	96.50%	5.30%	0.25	84.20%	91.20%	7.00%	0.125
Investigation (*n* = 27)	92.60%	100.00%	7.40%	-	63.00%	70.40%	7.40%	0.687
Knowledge (*n* = 32)	87.50%	90.60%	3.10%	1.00	71.90%	84.40%	12.50%	0.344
Management (*n* = 43)	86.05%	88.40%	2.35%	1.00	51.10%	74.40%	23.3%	0.052
Pharmacology (*n* = 32)	81.30%	93.80%	12.50%	0.125	62.50%	81.30%	18.80%	0.146

The *p*-value for the comparison between the two rounds is also provided for each instance.

## Discussion

Over an 18-month longitudinal evaluation, GPT-4 demonstrated significantly improved performance in answering UKMLA questions. This represents a significant improvement and suggests that the deployed GPT-4 model has become more capable at medical reasoning and clinical decision-making as assessed by this high-stakes examination standard.

However, GPT-4’s improvement cannot be attributed solely to the model’s autonomous learning. The consumer GPT interface does not provide access to dated model snapshots, and OpenAI deploys updates to the underlying GPT-4 model without public disclosure. The observed performance change, therefore, may reflect vendor-side model updates, changes in training data composition, modifications to alignment protocols, stochastic variation, or a combination of these factors. We cannot distinguish between these mechanisms based on available evidence.

Our findings are consistent with documented updates to GPT-4’s training and alignment pipeline, including iterative refinements to (1) the training datasets, (2) the underlying neural network parameters, and (3) the natural language understanding and generation mechanisms. GPT-4 has been shown to undergo iterative refinements involving retraining on updated datasets and reinforcement learning from human feedback (RLHF) (29). RLHF enables continual behavioral improvement by structuring human feedback from both expert labellers and real-world user interactions. Prior work has demonstrated that large language models can achieve substantial gains on complex reasoning tasks with chain-of-thought prompting, in which intermediate reasoning steps are elicited or internally modelled to support more accurate final answers (30). While such reasoning traces may not be explicitly exposed to end users, their incorporation into prompting strategies and model optimisation frameworks plausibly contributes to improved medical reasoning performance over successive model iterations. The primary driver of longitudinal performance change in GPT-4 is likely backend model and alignment modifications across releases, rather than autonomous learning from individual user interactions (32). Our observed improvement is therefore consistent with structured, vendor-managed updates to training and alignment pipelines.

There was a statistically significant improvement in GPT-4’s performance on questions with multiple-choice options compared to those with open-ended options. Studies have shown that multiple-choice questions (MCQs) support rote memorisation, whereas open-ended questions (OEQs) favour conceptual learning (33, 34). Answering MCQs requires reliance on pattern recognition and familiarity-based answer identification, whereas OEQs require active engagement, understanding, and the ability to derive information from a data source. Since GPT-4 demonstrates a significant increase in UKMLA questions when presented as MCQs, this indicates that its familiarity-based understanding has improved (35, 36). More needs to be done to prime and enable GPT-4 to better understand these concepts, so that knowledge is application-based rather than recognition-based.

Domain-specific analysis showed no notable increases across domains, which remained generally unchanged. As an inanimate entity, GPT-4 relies on building from its foundational models to improve its capabilities (37). Better performance results from further training and human feedback (38). This suggests that ongoing training and teaching of GPT-4 to foster the overall development of its abilities will lead to better clinical correlations and applications.

The pronounced trough and subsequent rise in management question accuracy under non-MCQ conditions reflect the higher cognitive burden of multi-step synthesis involved in clinical management. This could possibly be less well captured by pattern-recognition-based formats used by LLMs. In contrast, GPT-4 demonstrates near-ceiling performance on investigative questions using MCQ prompts. Across diagnoses, core medical knowledge and pharmacology, non-MCQ scores moved closer to MCQ scores, suggesting better recall and application without prompts in round two. By contrast, a persistent gap between MCQ and non-MCQ scores in management questions indicates that GPT-4’s improved clinical reasoning does not translate uniformly into all domains. This aligns with the literature, which highlights the importance of contrasting competing options as the foundation of clinical management, rather than relying solely on recall of facts (19, 39–41).

We used the publicly available UKMLA question paper published by the Medical Schools Council (MSC) on their official website. Two question papers with worked answers are accessible on the MSC site. Although it is challenging to identify the specific datasets used to train GPT-4, the availability of accurate answers to the questions we input should enable it to achieve a perfect 100% score on both papers at both time points, since we allowed web browsing. However, since this is not observed, hypotheses can be formulated to explain GPT-4’s performance in the UKMLA.

AI hallucination is a widely discussed concept within the AI scholarly community, which may help explain why GPT-4 has not achieved perfect scores on examinations. It is defined as the generation of fictional information based on associations formed around the most probable occurrence (42). Some critics of GAI have suggested that GPT-4 identifies a string of words that most closely relates to the question and coherently expresses it without any form of thinking or reasoning (43). Hallucinations are often classified into three categories: input, fact, and context, which can lead to the production of serious and incorrect content for the end user (38). Since medicine is evidence-based, a wide range of diverse information is available for nearly every medical condition, diagnosis, and treatment option (44). While it is the responsibility of the physician to understand the levels of scientific evidence and study strengths, and to incorporate this into their practice, the neural networks of GPT-4 may lack these capabilities, potentially explaining the gap between the expected and actual scores e of the GAI in the UKMLA (39, 45).

Mirroring is a concept rarely discussed in the literature, but it could help explain the gap between GPT-4’s theoretical score and its actual performance in the UKMLA. In GAI, mirroring refers to the way it perpetuates pre-existing human biases. Since GAIs are trained on datasets mainly sourced from humans, they naturally embody systemic conscious and unconscious biases and understandings that humans also experience (40). While these biases could lead to misinformation if amplified, they may also account for the differences seen in UKMLA results. Because human nature involves errors, developing a human-like LLM such as GPT-4 will inherently lead to human-like fallacies (19, 41, 46).

The results of this study indicate that GPT-4’s performance on the UKMLA question bank improved substantially over an 18-month period, reflecting iterative vendor-side updates to training data, alignment strategies, and model capabilities. While the consumer interface label remains “GPT-4”, the underlying model has likely evolved through backend refinements, as evidenced by the observed performance gains (47).

GPT-4 has shown significant enhancements in its capabilities within the UKMLA, with improvements across multiple specific domains. These findings support the inclusion of GPT-4 as a supplementary tool in medical education, where it could provide formatted explanations of clinical concepts or help identify knowledge gaps to inform personalised learning (48, 49). The significant improvements also suggest that GPT-4 has the potential to be utilised by clinicians as a decision-aiding tool or a source of patient information. However, performance evaluations of GAI triaging in real-world emergency scenarios have yielded heterogeneous and limited results. Hence, further training and evaluation of models in real-life scenarios are required before real-world implementation (50). Medical educators and clinicians considering GPT-4 integration should treat these findings as provisional support for pilot programmes with structured evaluation, while recognising clinical implementation requires substantially more evidence.

The development of retrieval-augmented generation (RAG) offers promise for improving both medical education and the potential clinical applications of GAI (51, 52). RAG provides a cost-effective method to optimise LLM outputs by enabling it to reference an external knowledge base before generating a response. Early studies have shown that RAG enables models to deliver significantly better, more consistent responses with fewer hallucinations. Supplying GAI with medical textbooks, institutional lecture notes, or exam-oriented datasets could give students a tailored, interactive chatbot to support their learning (53, 54). Likewise, providing national guidelines, peer-reviewed literature, or specific clinical pathways from national bodies or National Health Service (NHS) trusts could improve chatbot responses for clinicians and patients, making the chatbot a reliable reference tool. Based on our findings regarding GPT-4’s capabilities in this study, RAG pairings with GPT-4 could be used to deliver accurate, targeted information to users and, eventually, to develop customised learning plans based on individual learner characteristics to enhance students’ abilities.

While our study shows the rapidly evolving and improving capabilities of GAI, such as GPT-4, we should approach their integration into clinical and medical education with cautious optimism. Potential risks of continuing to use GPT-4 in medical training and clinical settings include: (1) a lack of understanding and interpretation of the information processed and generated, (2) the absence of ethical principles governing the use of GAI, (3) the spread of misinformation, and (4) an over-reliance on LLMs by students and medical professionals, which could weaken critical and lateral thinking skills. Therefore, resources such as GPT-4 should be used carefully, with users verifying the information provided (38, 55).

This study has several limitations. First, evaluations were conducted using the standard GPT-4 user interface without applying temperature optimisation, system-level prompt controls, or advanced prompt engineering techniques. While these strategies may improve model performance, they were intentionally excluded to reflect the typical usage patterns of lay clinicians and medical trainees, for whom such optimisations are not routinely accessible. Second, the analysis focuses on observable performance and knowledge drift over time rather than isolating specific backend mechanisms responsible for these changes, as model training details and update schedules remain opaque. Thirdly, results may not generalise to API-based deployments or alternative prompting configurations, where greater control over inference parameters and model snapshots is available. Fourthly, web browsing was enabled during testing; as the MSC mock papers are freely accessible online, the model may have retrieved question or answer content from the web rather than relying solely on internal clinical knowledge, potentially inflating accuracy. Finally, each question was administered only once, so within-model response variability was not captured (56).

## Conclusion

GPT-4’s performance in medical licensing exams shows promise in producing clinically capable GAI. Our longitudinal study of GPT-4’s results on the same UKMLA paper highlights notable improvements across categories, demonstrating the extensive developmental potential of these GAI in medicine. Institutions should consider using AI LLMs as educational aids to create learner-specific programmes that assist medical students. Any clinical application, such as a decision-support adjunct or patient information resource, should be considered provisional and would require substantially more validation before wider clinical deployment. However, caution is necessary when relying on these GAI, due to concerns such as (1) the dissemination of misinformation, (2) the teaching of incorrect concepts, and (3) the risk of creating de-skilled physicians through excessive dependence on GAI in clinical practice.

## Data Availability

The original contributions presented in the study are included in the article/Supplementary material, further inquiries can be directed to the corresponding author.

## Footnotes

1
https://chat.openai.com
