# Supplementary material for: The temporal changes in GPT-4 performance on UKMLA practice questions: educational and clinical implications
Source: Front Med (Lausanne). 2026 Jul 21;13:1888765. doi: 10.3389/fmed.2026.1888765 (PMC13434848; doi:10.3389/fmed.2026.1888765)
Supplement: Supplementary file 1 [file Table_2.DOCX]

| Category | Question stem | Correct answer | GPT-4’s answer |
| --- | --- | --- | --- |
| Correct | A 34 year old woman has sudden onset of right arm weakness and inability to speak. She has migraines and generalised joint pains. Four years ago, she had a deep vein thrombosis in her right leg.  Her pulse is 68 bpm and BP 178/94 mmHg. She has an expressive dysphasia. She has flaccid weakness of her right arm and facial droop on the right lower half of her face.  Investigations:  Haemoglobin 118 g/L (115–150)  White cell count 4.3 × 109/L (3.8–10.0)  Neutrophils 2.1 × 109/L (2.0–7.5)  Lymphocytes 0.6 × 109/L (1.1–3.3)  Platelets 132 × 109/L (150–400)  Total cholesterol 4.6 mmol/L (<5.0)  CT scan of head left frontoparietal infarct  What additional investigation is most likely to reveal the underlying cause of her stroke? | Correct answer: B) Anticardiolipin antibody | **‘Antiphospholipid antibody testing,** specifically: **Lupus anticoagulant, Anticardiolipin antibodies, Anti-β2 glycoprotein I antibodies’** |
| Incorrect | A 55 year old man attends the GP surgery concerned that he may be a carrier of cystic fibrosis. The condition has just been diagnosed in his 5 year old grandson. He has heard that this is an inherited condition, but no one else in his family has the illness.  What is the likelihood that the grandfather is a carrier? | Correct answer: A) 1 in 2 | ‘100% likelihood that he is a carrier’ |
| Indeterminate | A 50 year old man has had increasing breathlessness when climbing the stairs. He has no chest pain, wheeze or cough. He has COPD. He takes a salbutamol inhaler several times per day. He used to smoke until 3 months ago and has a 30 pack-year smoking history. His weight is stable and he is otherwise well. Investigation: FEV1 : 75% predicted What is the most appropriate next step in pharmacological management? | Correct answer: B) Inhaled tiotropium and salmeterol | ‘Add a long-acting muscarinic antagonist (LAMA), e.g. tiotropium’ |

**Supplementary Table S1**: Scoring rubric and exemplar responses for Non-MCQ responses
